# *SESN2* prevents the slow-to-fast myofiber shift in denervated atrophy via *AMPK/PGC-1α* pathway

**DOI:** 10.1186/s11658-022-00367-z

**Published:** 2022-08-09

**Authors:** Xiaofan Yang, Pingping Xue, Zhenyu Liu, Wenqing Li, Chuyan Li, Zhenbing Chen

**Affiliations:** 1 Department of Hand Surgery, Union Hospital, Tongji Medical College, Huazhong University of Science and Technology, Wuhan, 430022 China; 2 Department of Pharmacy, Tongji Hospital, Tongji Medical College, Huazhong University of Science and Technology, Wuhan, 430030 China; 3 Department of Hand and Foot Surgery, Union Shenzhen Hospital, Huazhong University of Science and Technology, Shenzhen, 518052 China

**Keywords:** Denervation, Skeletal muscle atrophy, Myofiber type transition, *SESN2*, AMPK/PGC-1α

## Abstract

**Background:**

Sestrin2 (*SESN2*), a stress-inducible protein, has been reported to protect against denervated muscle atrophy through unfolded protein response and mitophagy, while its role in myofiber type transition remains unknown.

**Methods:**

A mouse sciatic nerve transection model was created to evaluate denervated muscle atrophy. Myofiber type transition was confirmed by western blot, fluorescence staining, ATP quantification, and metabolic enzyme activity analysis. Adeno-associated virus (AAV) was adopted to achieve *SESN2 *knockdown and overexpression in gastrocnemius. *AMPK/PGC-1α* signal was detected by western blot and activated with 5-aminoimidazole-4-carboxamide ribonucleotide (AICAR). C2C12 myotubes with rotenone treatment were adopted for in vitro experiments.

**Results:**

*SESN2* was found to be upregulated in denervated skeletal muscles and rotenone-treated C2C12 cells. Knockdown of *SESN2* aggravated muscle atrophy and accelerated myofiber type transition from slow-twitch to fast-twitch. Moreover, *AMPK/PGC-1α* signaling was proven to be activated by *SESN2* after denervation, which further induced the expression of hypoxia-inducible factor *HIF2α*. Exogenous activation of *AMPK/PGC-1α* signaling could counteract the addition of slow-to-fast myofiber shift caused by *SESN2* knockdown and lead to the retainment of muscle mass after denervation.

**Conclusion:**

Collectively, the present study indicates that* SESN2* prevents myofiber type transition from slow-twitch to fast-twitch and preserves muscle mass in denervated atrophy via *AMPK/PGC-1α* signaling. These findings contribute to a better understanding of the pathogenesis of muscle atrophy and provide novel insights into the role of* SESN2* in myofiber type transition.

**Supplementary Information:**

The online version contains supplementary material available at 10.1186/s11658-022-00367-z.

## Introduction

Skeletal muscles primarily comprise long cylindric multinuclear cells, referred to as myofibers, which are responsible for muscular contractile activity [1]. Every skeletal muscle is composed of a heterogeneous collection of myofiber types with different physiologic adaptation in reaction to stimuli. The contraction of slow type-I myofibers is slow and fatigue-resistant, while fast myofibers contain a more efficient sarcoplasm reticulum and display faster contraction activity [2]. Every myofiber has a special molecule-level signature of myosin heavy-chain molecules (MHCs). Slow type-I myofibers predominantly express MYH7, but fast myofibers can be divided into three types: IIa (MYH2), IIx (MYH1), and IIb (MYH4). These fibers also differ in terms of oxidative/glycolytic metabolism, with type I and IIa fibers being more oxidative and type IIb fibers more glycolytic [3]. Muscle atrophy, characterized by a gradual decrease of myofiber transection area and protein levels, develops rapidly with remarkable weakness posterior to denervation and is related to the unsatisfactory prognoses of peripheral nerve damage [4]. Denervation-induced muscle atrophy is usually accompanied by a slow-to-fast shift in fiber type and MHC isoform profile, while the underlying mechanism has barely been elucidated [5].

The mutual transition of myofiber type is regulated by complex signaling pathways, in which the transcriptional co-activator* PGC-1α*, a key regulator of mitochondrial biogenesis, has been frequently reported [6]. During resistance and endurance exercise, the expression of *PGC-1α* achieves a remarkable increase, which induces the fast-to-slow myofiber shift and protects against muscle atrophy [7]. Furthermore, overexpression of *PGC-1α* in transgenic mice and pigs increased the proportion of red/oxidative type I fiber, accompanied by elevated levels of oxidative fiber markers such as MHC1, MHC2x, myoglobin, and Tnni1 [8]. As we know,* PGC-1α* is a downstream transcriptional regulator of AMP-activated protein kinase (*AMPK*, a key sensor of cellular metabolic and energy homeostasis), targeting *AMPK* to regulate myofiber type transition by various drugs is thus feasible [9, 10]. For example, naringin was reported to remarkably elevate the protein levels of slow MHC, myoglobin, and troponin I type I slow skeletal (troponin I-ss) and the activities of succinate dehydrogenase (SDH) and malate dehydrogenase (MDH), as well as reduce the fast MHC level and the activity of lactate dehydrogenase (LDH), accompanied by the activation of *AMPK/PGC-1α* signaling in mice and C2C12 myotubes [11].

Sestrins are stress-inducing proteins that prevent reactive oxygen species (ROS)- or endoplasmic reticulum stress (ERS)-related symptoms by inhibiting mTORC1 via the stimulation of* AMPK* [12–14]. Among three sestrin homologs (SESN1–3) in mammalian species, our team, in recent years, discovered that the expression level of *SESN2* in skeletal muscle is increased upon denervation. Moreover,* SESN2* was proven to mediate skeletal muscle accommodation to aberrant mitochondria function and ERS and was an endogenous attenuator of denervation-induced muscle atrophy [15]. However, whether* SESN2* plays a role in denervation-induced slow-to-fast myofiber shift remains unclear. Thus, the purpose of this study was to further elucidate the relationship between* SESN2* expression and myofiber type transition upon denervation. The results of this study will clarify a novel role and mechanism of* SESN2* in denervated muscle atrophy.

## Materials and methods

### Animal procedures

Male 10-week-old C57BL/6 J mice, kept in a 24 h light/dark period environment, were provided by SPF (Beijing) Biotechnology Co., Ltd. Denervation was completed via surgery on the right hind legs as delineated previously [16]. The mice were then randomly divided into four groups (six mice per group): sham operation group (control); denervation group; denervation + AAV-shSESN2 (adeno-associated virus, U6-MCS-CAG-EGFP; Genochem, China) group and denervation + AAV-*SESN2* group (CMV-betaGlobin-MCS-SV40 PolyA, Genochem). The sequence of shSESN2 primers was 5′-GCGTCTTTGGCATCAGATACG-3′. AAV9 injections of gastrocnemius (GAS) were completed 3 weeks in advance to construct *SESN2* knockout or overexpression models. In particular, 10 µl of virus (1.0 × 10^12^ viral genomes (vg)/ml) were injected into each point around the gastrocnemius. To prevent the backflow of viral particles, the syringe was left in place for an extra 5 min after injection. Four to six injections were conducted on every limb. As for preparation of *AMPK* activation models, AICAR (an *AMPK* activator; MedChemExpress, USA, HY-13417) was administered to the mice via an intraperitoneal injection at a dose of 500 mg/kg once a day for 7 days prior to operation. An additional 7 days of AICAR treatment was performed until muscles were collected. Mice were euthanized by cervical dislocation at the indicated time, and GAS, extensor digitorum longus (EDL), and soleus (SOL) were removed, weighed, and frozen for the next experiments. For cervical dislocation, a trained person grasped the skin on the back of the neck by the thumb and forefinger and immediately pulled on the base of the tail in an opposite upward direction from the head. The immediate dislocation of the spinal column from the brain ensured death within a few seconds.

All animal experiments were completed as per the guidelines of the Chinese National Institutes of Health and obtained the approval of the Ethical Committee on Animal Experiments (Huazhong University of Science and Technology, no. 2021-S2789).

### Muscle mass measurements, H&E staining, and fiber diameter quantification

Muscles (GAS, EDL, and SOL) of the denervated and control sides were collected and weighed at the proper temporal points. The wet weight ratio (muscular weight of the denervated side divided by the weight of the control side) was utilized to assess muscle atrophy.

For hematoxylin and eosin (H&E) staining, muscle samples were subjected to fixation in paraformaldehyde (4%) for 24 h, and afterwards subjected to dehydration and paraffin embedment. The 4-μm cross-cutting muscle sections were acquired before H&E staining (Bioyear, PRC) as per the instructions.

For quantitative analysis of fiber diameters, muscular transection was stained for wheat germ agglutinin (WGA) to visualize the fibers. ImageJ program was leveraged to quantify the minimum Feret’s diameter of myofibers stochastically selected within each group.

### Western blot

The primary antibodies below were utilized: myosin heavy chain (MHC; R&D Systems, USA, MAB4470), slow MHC (Abcam, ab185967), fast MHC (Abcam, ab91506), MHC IIa (Abcam, ab124937), MHC IIb (Abcam, ab221149), myoglobin (Santa Cruz, USA, sc-393020), troponin I-ss (Santa Cruz, sc-514899), TOM20 (Abcam, UK, ab186735), TIM23 (Abcam, ab230253), SESN2 (Abcam, ab178518), AMPK (Cell Signaling Technology [CST], USA, 5831), phospho-AMPK (CST, 50081), PGC-1α (Abcam, ab106814), HIF-2α (Abcam, ab109616), GAPDH (Protein Technology, PRC, 60004-1-Ig). The primary antibody dilution factors were 1:3000 (R&D Systems), 1:5000 (Protein Technology), or 1:1000 (Abcam and CST).

In short, the protein was abstracted via RIPA lysis buffering solution to which 1% protease suppressor was added (Roche, USA, 11206893001). The same quantity of protein (10–50 μg) was isolated via 10% SDS–PAGE, and afterwards moved onto nitrocellulose films (Merck Millipore, USA, Z358657). The films were subjected to blockade with 5% w/v BSA prior to cultivation with the first antibodies under 4 °C overnight. Subsequently, the films were cultivated with second antibodies conjugated with antirabbit or antimouse IgG (Abcam) for 60 min under ambient temperature and visualized via the Immobilon ECL matrix tool (Merck Millipore, WBKLS0050).

### Immunofluorescence

Paraffin-embedded GAS underwent cross-sectional cuts, which were made into 4-μm sections as previously stated [16]. Following antigen retrieval, permeabilization, and goat serum blocking, primary antibody (mouse anti-slow MHC antibody and rabbit-anti-fast MHC antibody; Abcam) incubation was implemented at 4 °C. Next, samples were stained for 1 h using secondary Alexa Fluor 488-conjugated goat anti-mouse and Alexa Fluor 647-conjugated goat anti-rabbit IgG antibodies (Abcam, ab150113 and ab150079), followed by 5 min DAPI/PI (Sigma, USA, D9542) staining. The samples were then imaged with a fluorescence microscope. Similar processes were oriented with C2C12 cells.

### ATP level

ATP contents in mouse GAS were identified via ATP analysis tool (Beyotime, China, S0026). Briefly, GAS tissue was homogenized in ATP assay lysis buffering solution, and the supernatant was harvested via centrifugation at 12,000*g* for 300 s under 4 °C and subjected to quantification via BCA analysis. Subsequently, 100 μl ATP identification reagent was supplemented into 100 μl supernatant, and the firefly luciferase (FFL) activity was identified and studied via luminescent spectrometric analysis (EnSpire, USA). The ATP content was normalized to cell protein level and presented as proportion (%) in contrast to the controls. Similar procedures were performed in C2C12 cells.

### Metabolic enzyme activity analysis

The activities of LDH and SDH were identified via commercially available tools bought from Nanjing Jiancheng Biological Engineering Institution (China, A020-1-2 and A022-1-1) as per the supplier’s specification. Specific enzyme activity was presented as U/mg protein.

### Cell culture and transfection

C2C12 cells were obtained from iCell Bioscience Inc. and cultivated in DMEM (Gibco, USA) including 10% FBS (Gibco), 100 units/mL penicillin, and 100 μg/mL streptomycin solution (Sangon Biotech, China) in 5% CO_2_ at 37 ℃ in a cell culture incubator. Cells were differentiated into myotubes posterior to the cultivation with differentiation medium (2% horse serum in DMEM) for 1 week as delineated previously [11]. For genetic knockout, three short interference RNAs for murine* SESN2* (siSESN2) and negative controls (siNC) were synthesized by RiboBio Company (PRC). The transfection process was completed as per the specification of Lipofectamine 2000 (Invitrogen, USA, 11668019). Cells were collected at 48 h posterior to the transfection process for the subsequent assays. siRNAs with maximal suppressive effect were utilized for assays later on. The siRNA sequences are presented in Additional file 1: Table S1.

### Detection of mitochondrial ROS level

Identification of mitochondrial ROS (mtROS) was completed via the fluorogenic stain MitoSOX Red (Thermo Fisher, USA, M36008), which was targeted to mitochondria in alive cells. In short, 5 mM stock solution and 5 μM work solution of MitoSOX were produced as per the supplier’s specification. Cells were cultivated in work solution for 600 s without light under 37 °C and afterwards cleaned three times in a warm buffering solution. The pictures of alive cells were collected via a confocal laser scan microscope. Finally, more than 30 cells in every group were chosen stochastically to produce the eventual outcomes.

### Statistical analysis

Statistical analysis of three independent experiments with at least three technical repetitions was conducted using GraphPad Prism 9.0.0 (GraphPad, USA), and the statistical data were expressed as mean ± standard deviation. Differences were evaluated by one-way or two-way analysis of variance (ANOVA) with Tukey post-hoc test. *P*-value less than 0.05 was considered statistically significant.

## Results

### Denervation led to skeletal muscle atrophy and slow-to-fast myofiber shift

**Fig. 1:**
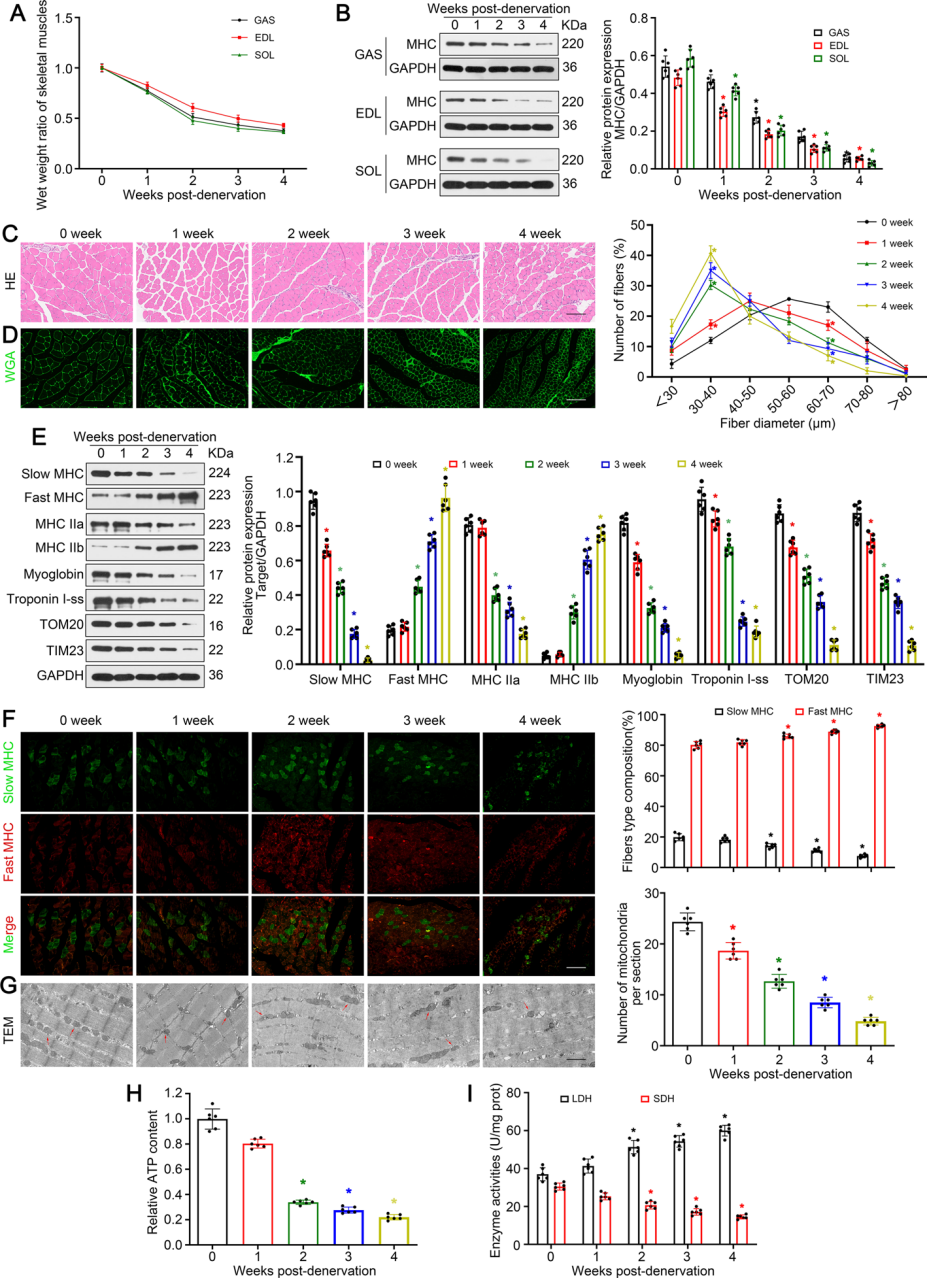
Denervation led to skeletal muscle atrophy and slow-to-fast myofiber shift. **A** Wet weight ratio (the weight of the operational side divided by the contralateral side) of GAS, EDL, and SOL at indicated timepoints post-denervation. **B** Western blot analysis of the dynamic changes of MHC protein expression after denervation. **C**, **D** Analysis of gastrocnemius fibers diameter by H&E and immunofluorescence staining. Scale bar, 50 μm. **E** Western blot analysis of proteins related to muscle fiber type. **F** Immunofluorescence analysis of the fiber type composition in denervated GAS. Scale bar, 50 μm. **G** Microstructure of GAS. Red arrows indicate mitochondria. Scale bar, 2 μm. **H**, **I** Relative ATP content and metabolic enzyme activity (LDH and SDH) in GAS at different timepoints post-denervation. Data presented as mean ± standard deviation (SD). *n* = 6. *P < 0.05 versus control (week 0)

To reveal the development of denervation-induced muscle atrophy, a murine sciatic nerve cross-section model was constructed and diverse types of skeletal muscle were studied. As described previously [15], muscular mass of GAS (mixed-type muscle), extensor digitorum longus (EDL; fast-twitch muscles involving type IIb and IIx myofibers), and soleus (SOL; slow-twitch muscles involving type I and IIa myofibers) reduced quickly upon denervation. In this process, muscle atrophy exhibited a representative two-phase characteristic with rapid weight loss (approximately 47%) in 14 days at the beginning and afterwards a slighter reduction (approximately 14%) in the following 14 days (Fig. 1A). As a vital structure protein in myofiber, MHC was identified via western blot and unveiled a similar change as reported previously (Fig. 1B). Later, H&E staining and WGA immunofluorescence were performed to quantify fiber diameter, revealing that denervation drove the myofibers to decrease with a progressively smaller diameter (Fig. 1C, D). Overall, skeletal muscles, irrespective of muscle type, atrophied quickly upon denervation.

Muscle atrophy is usually accompanied by myofiber type transition. As seen in Fig. 1E, a significant decrease of slow MHC, MHC IIa, myoglobin, and troponin I-ss was observed over time after denervation. In contrast, fast MHC and MHC IIb, meanwhile, increased dramatically. Immunofluorescence analysis indicated similar results, showing decreased percentage of slow oxidative fibers and increased percentage of fast glycolytic fibers (Fig. 1F). As slow oxidative fibers contain more mitochondria than fast glycolytic fibers, such changes further led to reduced mitochondria number and ATP content (Fig. 1E, G, H). In addition, the activity of SDH and LDH, which were used as markers of oxidative and glycolytic capacities, was further detected. Obviously, denervation led to attenuated SDH activity and enhanced LDH activity (Fig. 1I). Overall, denervation promoted myofiber type transition from slow-twitch to fast-twitch.

### *SESN2* protected against muscle atrophy and myofiber type transition after denervation

**Fig. 2:**
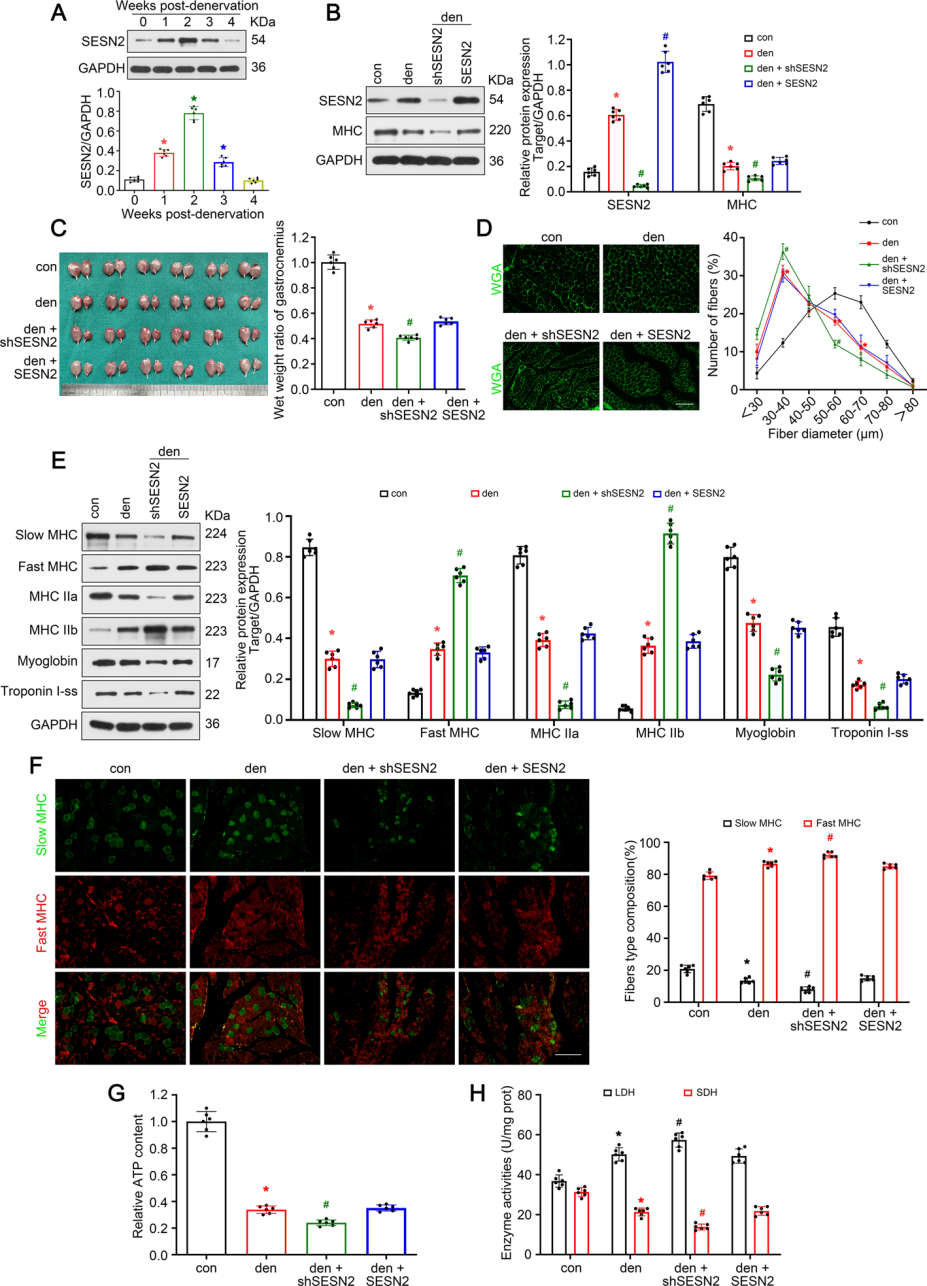
*SESN2* protected against muscle atrophy and myofiber type transition after denervation. **A** Western blot analysis of *SESN2* expression in GAS post-denervation. **B** Western blot analysis confirming the expression of* SESN2* and* MHC*. **C** Appearance and wet weight ratio of GAS in different groups. **D** Quantification of muscle fiber diameter by immunofluorescence staining of WGA. Scale bar, 50 μm. **E** Western blot analysis of proteins related to muscle fiber type. **F** Immunofluorescence analysis of fiber type composition in denervated GAS. Scale bar, 50 μm. **G**, **H** Relative ATP content and metabolic enzyme activities (LDH and SDH) in GAS. Data presented as mean ± SD. *n* = 6. *P < 0.05 versus control (week 0 or sham operation group). ^#^*P* < 0.05 versus denervation group. *Den* denervation, *Con* control

In this section, we explore the role of *SESN2* in denervated muscle atrophy and myofiber type transition. Firstly, evident accumulation of* SESN2* protein was identified in the 14 days at the beginning after denervation, before a progress decrease in the following 14 days (Fig. 2A). Given that the progression of muscle atrophy occurred mainly in the first 14 days in a coincidental manner, we speculated that *SESN2* might participate in the process of muscle atrophy. Subsequently, *SESN2* knockdown and overexpression models were produced to explore the participation of *SESN2* in the muscle atrophy of denervated GAS. The expression of* SESN2* was verified via western blot (Fig. 2B). GAS specimens were harvested 14 days after denervation. *MHC* expression and muscle mass evaluation unveiled that muscle atrophy was remarkably deteriorated posterior to* SESN2* knockdown and was mildly reversed via *SESN2* overexpression (not significant) (Fig. 2B, C). Analysis of fiber size revealed that the diameter of fibers decreased in denervated GAS and deteriorated by AAV-shSESN2 (Fig. 2D). Finally, the effect of *SESN2* on myofiber type transition was explored. As seen in Fig. 2E, F, knockdown of *SESN2* significantly accelerated the reduction of slow oxidative fiber and addition of fast glycolytic fiber. Overexpression of *SESN2* in denervated GAS exerted no obvious effect on myofiber type transition. ATP content and enzyme activity analysis revealed a trend consistent with the above data (Fig. 2G, H). Together, the above results indicate that* SESN2* protected against muscle atrophy and the slow-to-fast myofiber type shift upon denervation.

### Rotenone induced slow-to-fast fiber type shift in C2C12 myotubes

**Fig. 3:**
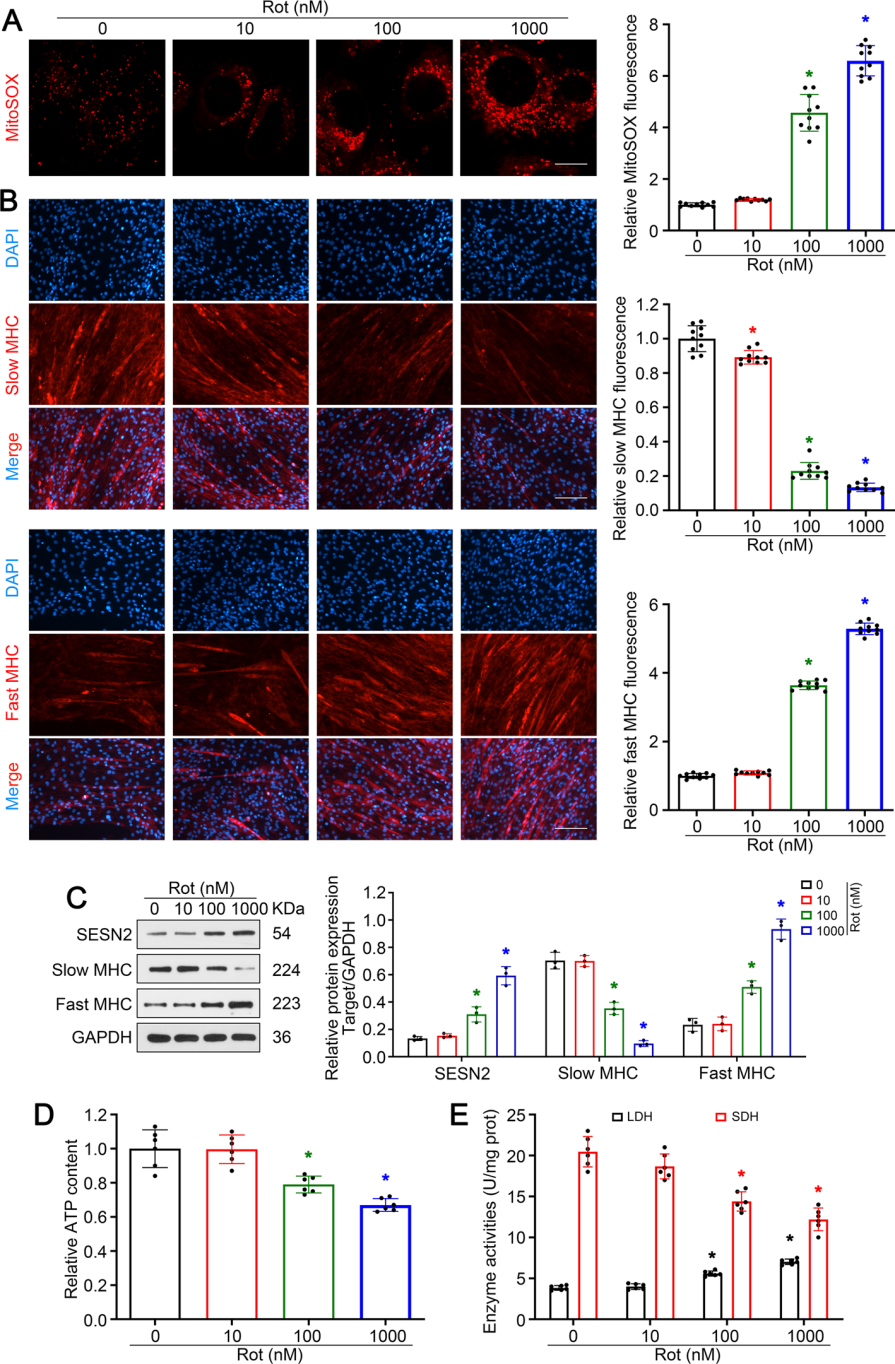
Rotenone induced slow-to-fast fiber type shift in C2C12 myotubes. **A** Mitochondrial ROS levels in C2C12 cells were detected by MitoSOX Red labeling. Scale bar, 10 μm. **B** Immunofluorescence analysis of slow and fast MHC. Scale bar, 50 μm. **C** Western blot analysis of slow and fast MHC. **D**, **E** Detection of relative ATP content and metabolic enzyme activity (LDH and SDH) in C2C12 myotubes. Data presented as mean ± SD. *P < 0.05 versus control (0 nM rotenone treatment). *Rot* rotenone

To replicate the denervated condition in vitro, C2C12 cells were treated with a concentration gradient of rotenone (a mitochondrial electron transport chain complex I inhibitor). As expected, obvious accumulation of mtROS was observed in C2C12 cells treated with 100 nM or more rotenone (Fig. 3A).* SESN2* was also induced by rotenone treatment (Fig. 3C). More importantly, rotenone treatment induced a significant reduction of slow MHC-positive myotubes and addition of fast MHC-positive myotubes (Fig. 3B). Analysis of protein expression, ATP content, and enzyme activity also revealed a consistent trend (Fig. 3C–E). Briefly, these data indicate that rotenone treatment could induce the slow-to-fast fiber type shift in C2C12 myotubes, which was similar to the in vivo denervation model. In the following experiments, 100 nM rotenone was adopted.

### Knockdown of *SESN2* accelerated slow-to-fast fiber type shift in rotenone-treated C2C12 myotubes

**Fig. 4:**
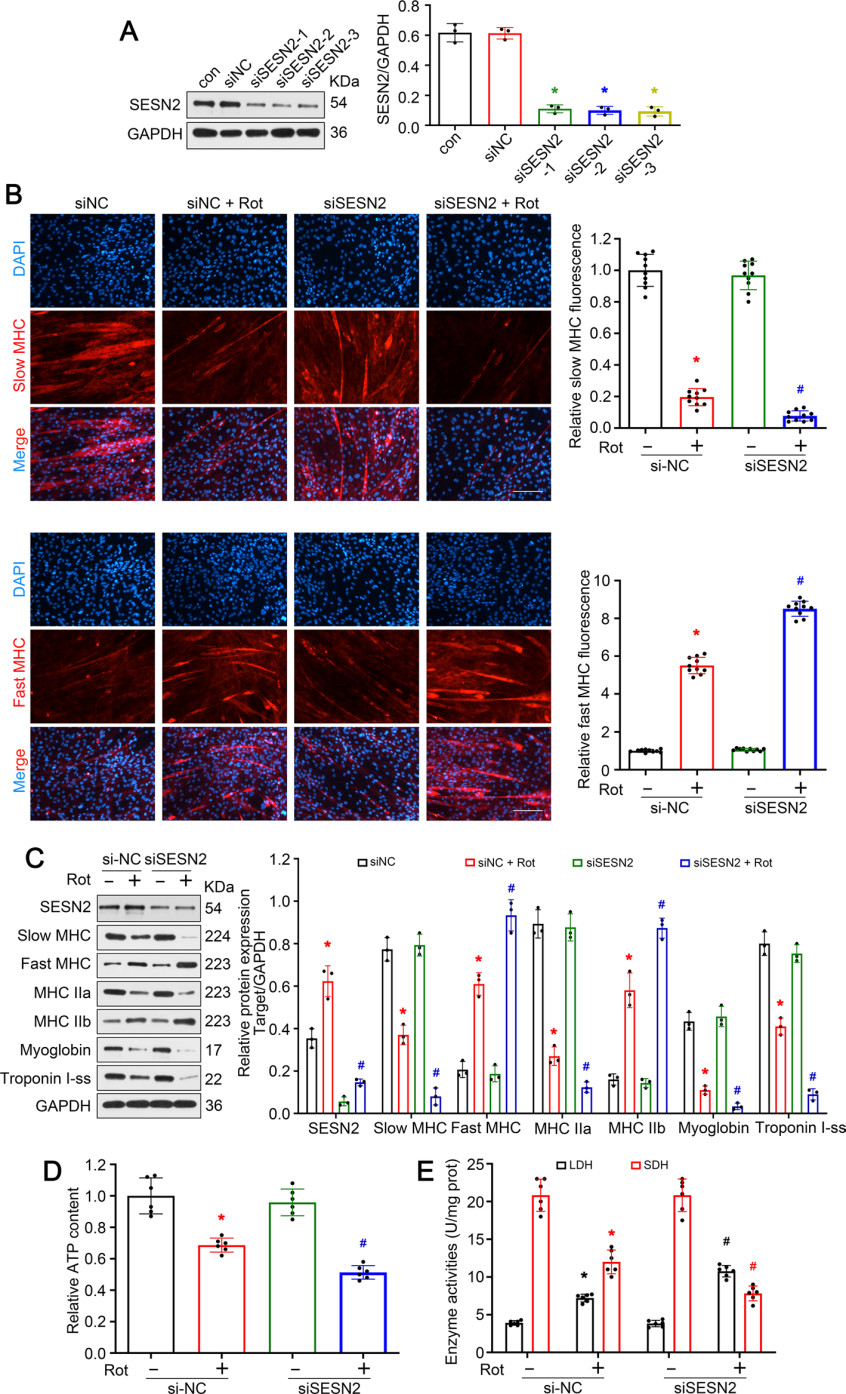
Knockdown of *SESN2* accelerated slow-to-fast fiber type shift in rotenone-treated C2C12 myotubes. **A** Western blot analysis confirming the effect of siSESN2. **B** Immunofluorescence analysis of slow and fast MHC. Scale bar, 50 μm. **C** Western blot analysis of proteins related to muscle fiber type. **D**, **E** Detection of relative ATP content and metabolic enzyme activities (LDH and SDH) in C2C12 myotubes. Data presented as mean ± SD. *P < 0.05 versus siNC group. ^#^*P* < 0.05 versus rotenone treatment group. *Rot* rotenone

To further confirm the role of* SESN*2 in myofiber type transition in vitro, knockdown of *SESN2* was achieved in rotenone-treated C2C12 cells. The inhibitory effect of siSESN2 was confirmed by western blot (Fig. 4A). Immunofluorescence analysis revealed that siSESN2 treatment significantly accelerated the rotenone-induced reduction of slow MHC-positive myotubes and addition of fast MHC-positive myotubes (Fig. 4B). The results of western blot indicated that knockdown of *SESN2* further suppressed the expressions of slow oxidative fiber-related proteins (slow MHC, MHC IIa, myoglobin, and troponin I-ss) and enhanced the expression of fast glycolytic fiber-related proteins (fast MHC, MHC IIb) (Fig. 4C). ATP content and enzyme activity detection displayed similar changes (Fig. 4D, E). These data prove that* SESN2* was required to prevent slow-to-fast fiber type shift in rotenone-treated C2C12 myotubes.

### *SESN2* prevented slow-to-fast shift in rotenone-treated C2C12 myotubes via *AMPK/PGC-1α* pathway

**Fig. 5:**
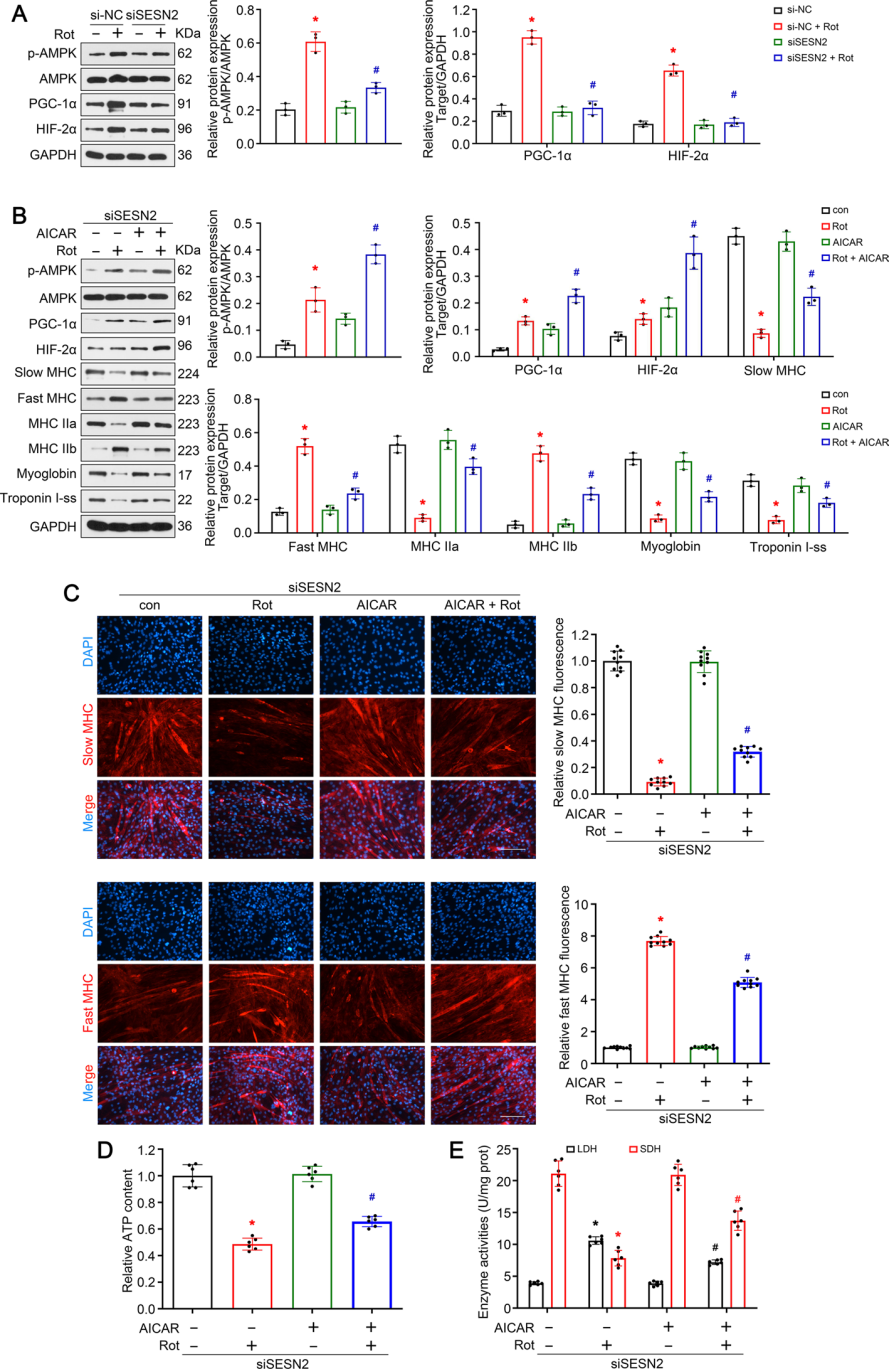
*SESN2* prevented slow-to-fast shift in rotenone-treated C2C12 myotubes via *AMPK/PGC-1α* pathway. **A** Western blot analysis of the regulatory effect of* SESN2* on the activity of *AMPK/PGC-1α* signaling. **B** Western blot analysis of C2C12 myotubes treated with rotenone and AICAR (an *AMPK* activator) to confirm the role of *AMPK/PGC-1α* signaling in myofiber type transition. **C** Immunofluorescence analysis of slow and fast MHC. Scale bar, 50 μm. **D**, **E** Detection of relative ATP content and metabolic enzyme activity (LDH and SDH) in C2C12 myotubes. Data presented as mean ± SD. *P < 0.05 versus control or siNC group. ^#^*P* < 0.05 versus rotenone treatment group. *Rot* rotenone, *Con* control

Given the vital role of *AMPK/PGC-1α* signaling in myofiber type transition, we wonder whether this signal was working downstream *SESN2*. As seen in Fig. 5A, rotenone treatment activated and induced expression of *PGC-1α* and* HIF-2α*, while knockdown of *SESN2* reversed the above variation, confirming the regulatory effect of *SESN2* on* AMPK/PGC-1α* signaling. To further clarify the role of *AMPK/PGC-1α* in myofiber type transition, C2C12 myotubes were pretreated with AICAR, an* AMPK *activator, before rotenone. It was seen that AICAR successfully activated *AMPK* and downstream* PGC-1α* and *HIF-2α*. Besides, AICAR treatment reversed the effect of rotenone, showing increased slow oxidative fiber-related proteins (slow MHC, MHC IIa, myoglobin, and troponin I-ss) and decreased fast glycolytic fiber-related proteins (fast MHC, MHC IIb) (Fig. 5B). Consistently, AICAR treatment reversed the rotenone-induced reduction of slow MHC-positive myotubes and addition of fast MHC-positive myotubes (Fig. 5C). This trend was further confirmed in the detection of ATP content and enzyme activity (Fig. 5D, E). To sum up, *AMPK/PGC-1α *signaling mediated the inhibitory effect of *SESN2* on myofiber type transition.

### *SESN2* activated* AMPK/PGC-1α *signaling in denervated muscle

**Fig. 6:**
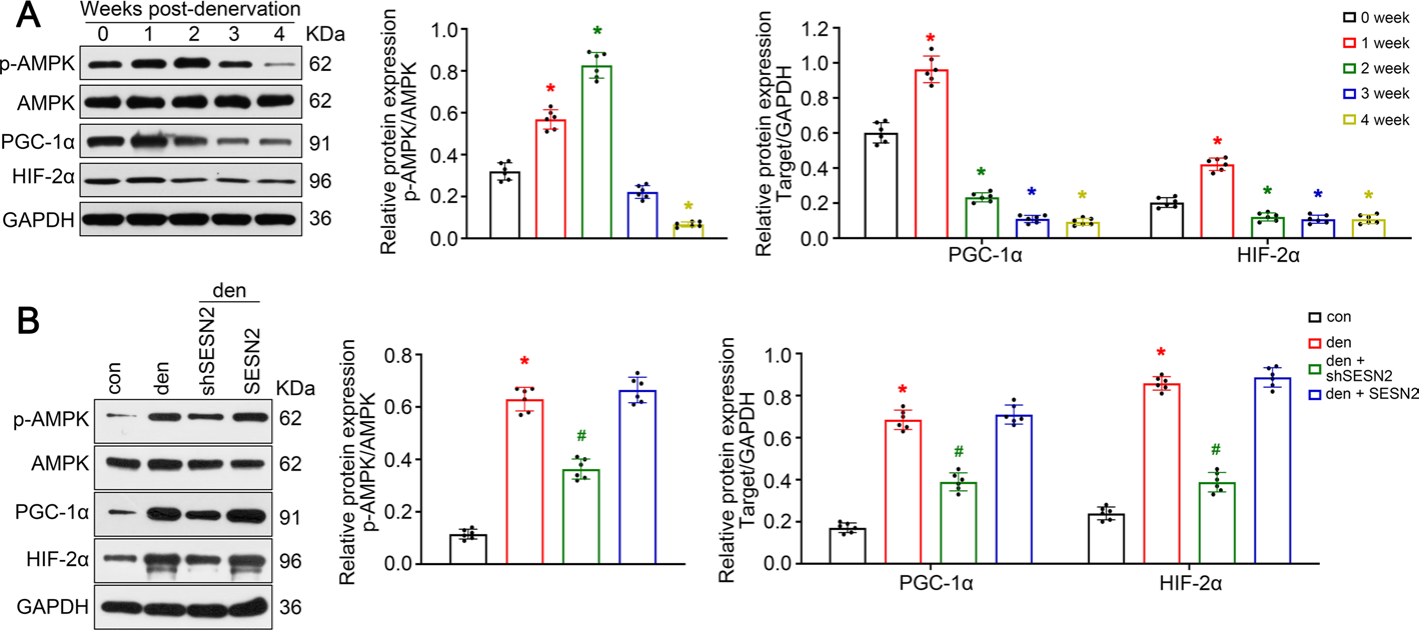
*SESN2* activated *AMPK/PGC-1α* signaling in denervated muscle. **A** Dynamic monitoring of *AMPK/PGC-1α *signaling in GAS post-denervation. **B** Western blot analysis confirming the regulatory effect of *SESN2* on the activity of *AMPK/PGC-1α* signaling. Data presented as mean ± SD. *n* = 6. *P < 0.05 versus control (week 0 or sham operation group). ^#^*P* < 0.05 versus denervation group. *Den* denervation, *Con* control

To investigate the relationship between *SESN2 *and *AMPK/PGC-1α* signaling in denervated atrophy, dynamic monitoring of protein content was performed. Similar to *SESN2*, *AMPK *was activated in the first 2 weeks after denervation, while the expression of *PGC-1α* and downstream *HIF-2α* reached a peak 1 week post-denervation and fell down rapidly afterwards (Fig. 6A). As *AMPK/PGC-1α* signaling is well documented in myofiber type transition, we speculated that this variation might be an endogenous protective response in the early stage of denervated muscle atrophy. Subsequently, GAS with* SESN2* knockdown was isolated 1 week post-denervation for western blot. As seen in Fig. 6B, knockdown of *SESN2* suppressed the activation of* AMPK/PGC-1α* in the early stage of denervation. Together, the above data prove that the *AMPK/PGC-1α *signaling was activated by *SESN2* in denervated muscle.

### *AMPK/PGC-1α *signaling prevented slow-to-fast myofiber shift and protected against muscle atrophy upon denervation

**Fig. 7:**
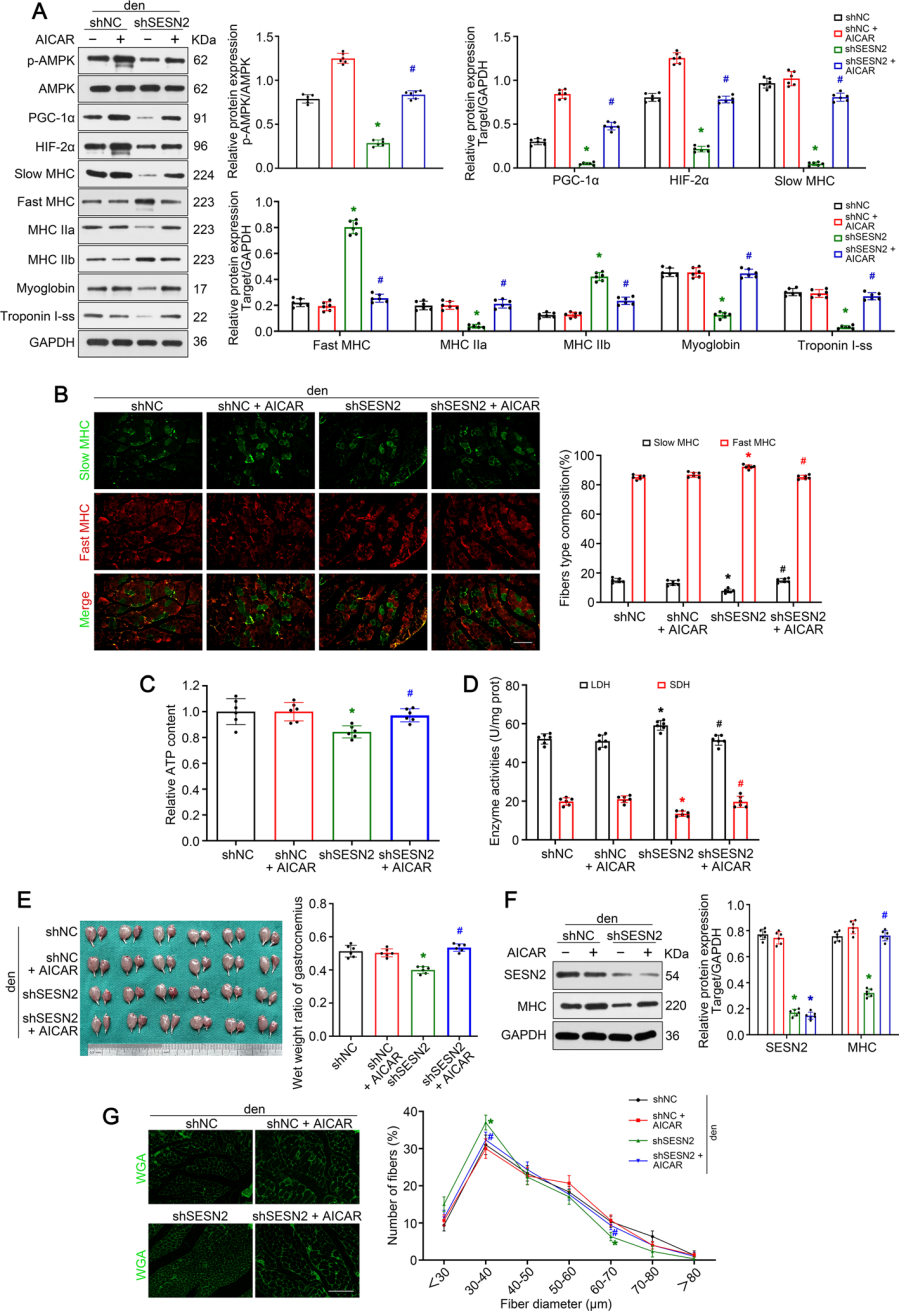
*AMPK/PGC-1α *signaling prevented slow-to-fast myofiber shift and protected against muscle atrophy upon denervation. **A** Western blot analysis confirmed the role of *AMPK/PGC-1α* signaling in myofiber type transition in denervated GAS. **B** Immunofluorescence analysis of fiber type composition in denervated GAS. Scale bar, 50 μm. **C**, **D** Relative ATP content and metabolic enzyme activities (LDH and SDH) in GAS with different treatment. **E** Appearance and wet weight ratio of GAS in different groups. **F** Western blot analysis confirmed the expressions of *SESN2* and *MHC*. **G** Quantification of muscle fibers diameter by immunofluorescence staining of WGA. Scale bar, 50 μm. Data presented as mean ± SD. *n* = 6. *P < 0.05 versus denervation + shNC group. ^#^*P* < 0.05 versus denervation + shSESN2 group. *Den* denervation

Here we wonder whether *AMPK/PGC-1α* signaling played a role in myofiber type transition. Western blot analysis of GAS isolated 1 week post-denervation indicated that knockdown of *SESN2* significantly accelerated the decrease of slow oxidative fiber-related proteins (slow MHC, MHC IIa, myoglobin, and troponin I-ss) and increase of fast glycolytic fiber-related proteins (fast MHC, MHC IIb) induced by denervation, while the AICAR treatment made up for the lack of* SESN2* (Fig. 7A). Consistently, knockdown of *SESN2 *led to a further reduction of slow oxidative fiber and addition of fast glycolytic fiber compared with the denervation + shNC group, while AICAR treatment reversed this trend induced by *SESN2* knockdown (Fig. 7B). Detection of ATP content and enzyme activity also consolidated the effect of AICAR (Fig. 7C, D). It needs to be emphasized that the analysis of fiber type, ATP content, and enzyme activity was performed in GAS 2 weeks post-denervation given that muscle atrophy happens more slowly than signal change. In short, these results demonstrate that *AMPK/PGC-1α* signaling attenuated the slow-to-fast fiber shift in denervated muscle.

In addition to the role of* AMPK/PGC-1α* signaling in myofiber type transition, its effect on muscle atrophy was further clarified. It was obvious that knockdown of *SESN2 *further aggravated the denervation-induced weight loss, MHC degradation, and fiber diameter reduction. Interestingly, the addition of AICAR reversed all these effects and achieved a similar degree of atrophy to that of the denervation + shNC group (Fig. 7E–G). The above results indicate that *AMPK/PGC-1α *signaling protected against muscle atrophy upon denervation.

## Discussion

According to previous research [17, 18], *SESN2* is constantly triggered upon various stresses, and exhibits cytoprotection features via regulating the synthetic process of proteins, autophagic activity, and apoptosis. In our previous research, ERS and mitochondrial dysfunction were found in denervated muscle, while the endogenous expression of* SESN2* in the early stage of denervation mediated unfolding protein response and mitophagy and ultimately alleviated muscle atrophy [15]. Given that myofiber type transition is a main feature of skeletal muscle atrophy, we wonder whether* SESN2* was involved therein. As expected, the present study indicated that *SESN2 *was induced soon after denervation and subsequently activated the* AMPK/PGC-1α *signal, which was shown to prevent the slow-to-fast myofiber type shift in denervated muscle. This finding provides new insight into the function of* SESN2* and extends our understanding of the process of myofiber type transition post-denervation.

In the past few years, myofiber type transition was observed and studied mainly in sports medicine, in which the *PGC-1α* signal was identified as the key regulator therein [19, 20]. As a versatile transcription co-activator,

*PGC-1α *participates in various cell activities, including adaptive calorigenesis, fatty acid oxidation, gluconeogenesis, and mitochondrial biogenesis [21]. In skeletal muscle,* PGC-1α *is highly expressed in slow oxidation fibers rather than fast glycolytic fibers [22]. Numerous studies have confirmed a close association of muscular contraction activity with elevated expressing level of *PGC-1α* [23, 24]. Endurance exercise triggers the expression of *PGC-1α* mRNA and protein in rats and humans. Moreover, overexpression of *PGC-1α* can reinforce mitochondria biogenesis and facilitate the fast-to-slow myofiber transition in cultivated myoblasts and transgenic mice, which improves exercise performance [25]. However, the mechanism underlying myofiber fiber transition in denervated muscle was rarely reported. In the present research, a new role of *PGC-1α* was identified, where *PGC-1α* acted as an endogenous protective molecular against the slow-to-fast fiber shift and ultimately delayed the process of denervated muscle atrophy.

HIF-2α, as a member of the the Per–ARNT–Sim–bHLH family of transcription factors that regulate the cellular response to hypoxic conditions [26, 27], was also detected in the present study. According to previous research, *HIF-2α* acts as a key mediator of *PGC-1α*-dependent myofiber type transition, a process that is partially muscle selective [22]. In our research, a similar expression pattern of *HIF-2α* and *PGC-1α* was observed in denervated GAS and C2C12 myotubes. Moreover, indirect upregulation of *PGC-1α* and *HIF-2α* prevented the slow-to-fast myofiber type shift and protected against muscle atrophy. Consistent with previous research, we speculate that *PGC-1α* regulation of myofiber type transition in denervated muscle is *HIF-2α *dependent.

In the dynamic monitoring of *AMPK/PGC-1α* signaling, we noticed a mismatch in the expression of *AMPK* and downstream *PGC-1α* and *HIF-2α*, as described in Fig. 6A. Given that the *SESN2–AMPK* pathway has been widely reported to activate autophagy and suppress protein synthesis [28–30], we speculate that the regulation of *SESN2–AMPK *pathway on* PGC-1α* expression is biphasic, with a promotion effect at early stage and a suppression effect afterward. Of course, other mechanisms might also be involved in *PGC-1α* regulation therein and worthy of further research.

Considering that different muscles contain different fibers and may respond differently to denervation [31], it will be difficult to apply this new regulatory mechanism to muscles other than GAS. As reported in previous research, type I myofibers of rat soleus underwent remarkable atrophy upon denervation, while type I myofibers in EDL maintained basically the same size in the first 14 days posterior to sciatic nerve section. A similar diversity was identified in type IIa myofibers, which underwent evident atrophy in soleus but merely minor atrophy in EDL. In rat diaphragm, denervation was similarly discovered to induce atrophy of type IIx and IIb myofibers, and showed no variation in type IIa and hypertrophy of type I myofibers [32]. However, the present study was meaningful in that it identified potential molecular pathological mechanisms of myofiber type transition upon denervation, which were previously unknown and out of the spotlight.

Here we noticed that overexpression of *SESN2* made no difference to myofiber type transition and muscle atrophy upon denervation. As stated before, we speculate that the induction of *SESN2 *in denervated gastrocnemius is an adaptive response that critically mediates skeletal muscle adaptation to denervation by preventing the slow-to-fast myofiber shift, and is not enough to counteract the denervation-induced muscle atrophy.

As the atrophy of skeletal muscle upon denervation is a complicated process, no suitable in vitro model has yet been designed capable of replicating such a denervated state. Given that mitochondrial dysfunction and ROS overproduction were considered as important features of denervated atrophy [16, 33], C2C12 cells with rotenone treatment were adopted in the present research and achieved similar characteristics (evidenced by increased accumulation of ROS and obvious trend of slow-to-fast fiber shift). In addition, only male mice were adopted in this research in order to avoid the potential influence of biological sex on myofiber type and muscle atrophy. Considering that the skeletal muscle kinetics and fiber type composition was quite different in male and female mice [34], the process of muscle atrophy in female mice requires further study.

## Conclusions

The present research revealed a new role of *SESN2* in denervated muscle atrophy, where *SESN2 *activated the* AMPK/PGC-1α* signal at an early stage of denervation and prevented the slow-to-fast myofiber shift. These findings expand our understanding of *SESN2* in muscle atrophy, and provide potential regulatory targets for myofiber type transition.

## Supplementary Information

**Additional file 1: Table S1.** The sequences of siRNAs.

## Data Availability

The datasets used and/or analyzed during the current study are available from the corresponding author on reasonable request.

## Abbreviations

*SESN2*: Sestrin2
MHC: Myosin heavy chain
AMPK: AMP-activated protein kinase
Troponin I-ss: Troponin I type I slow skeletal
SDH: Succinate dehydrogenase
LDH: Lactate dehydrogenase
ROS: Reactive oxygen species
ERS: Endoplasmic reticulum stress
GAS: Gastrocnemius
EDL: Extensor digitorum longus
SOL: Soleus
WGA: Wheat germ agglutinin
