# Supplementary material for: SESN2 prevents the slow-to-fast myofiber shift in denervated atrophy via AMPK/PGC-1α pathway
Source: Cell Mol Biol Lett. 2022 Aug 9;27:66. doi: 10.1186/s11658-022-00367-z (PMC9361691; doi:10.1186/s11658-022-00367-z)

Additional Materials for

**SESN2 prevents the** **slow-to-fast myofiber shift in denervated atrophy via** **AMPK/PGC-1α pathway**

Xiaofan Yang^1,#^ · Pingping Xue^2,#^ · Zhenyu Liu^1^ · Wenqing Li^3^ · Chuyan Li^3^ · Zhenbing Chen^1,^*

^1^Department of Hand Surgery, Union Hospital, Tongji Medical College, Huazhong University of Science and Technology, Wuhan 430022, China

^2^Department of Pharmacy, Tongji Hospital, Tongji Medical College, Huazhong University of Science and Technology, Wuhan 430030, China

^3^Department of Hand and Foot Surgery, Union Shenzhen Hospital, Huazhong University of Science and Technology, Shenzhen 518052, China

^#^These authors contributed equally to this work

*Corresponding author: Zhenbing Chen, MD

Tel: +86 13871103730

Fax: +86 02785351628

E-mail address: zbchen@hust.edu.cn

**Additional Tables**

**Table S1. The sequences of siRNAs.**

| **SiRNA Targets** | **Sequences** |
| --- | --- |
| siSESN2#1 sense | 5′- GCGCTTTCATTCCAGTGGAAGAGAT-3′ |
| siSESN2#2 sense | 5′- GATGACTATGATTACGGCGAGGTAA-3′ |
| siSESN2#3 sense | 5′- CAGAGAAGGTTCATGTGAACTTGCT-3′ |
| siNC sense | 5′- CAGGGAAACTTGTGTTCAATGAGCT-3′ |

**Raw images for western blots**


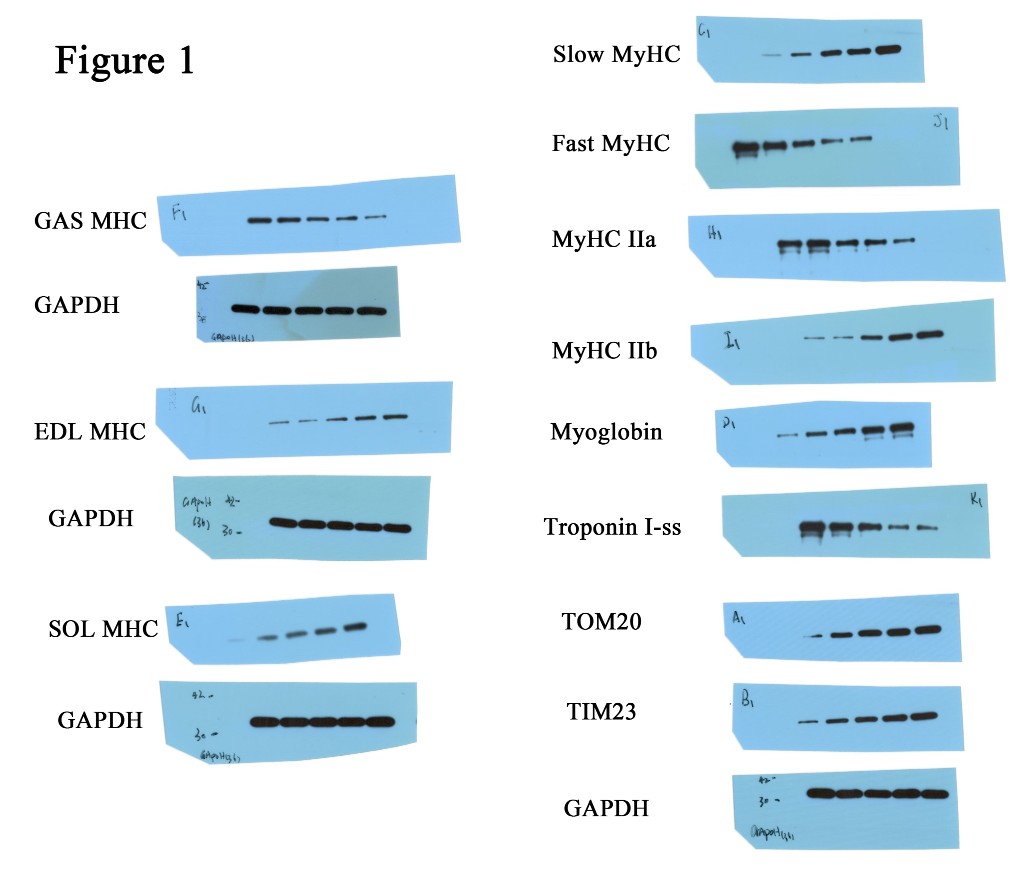


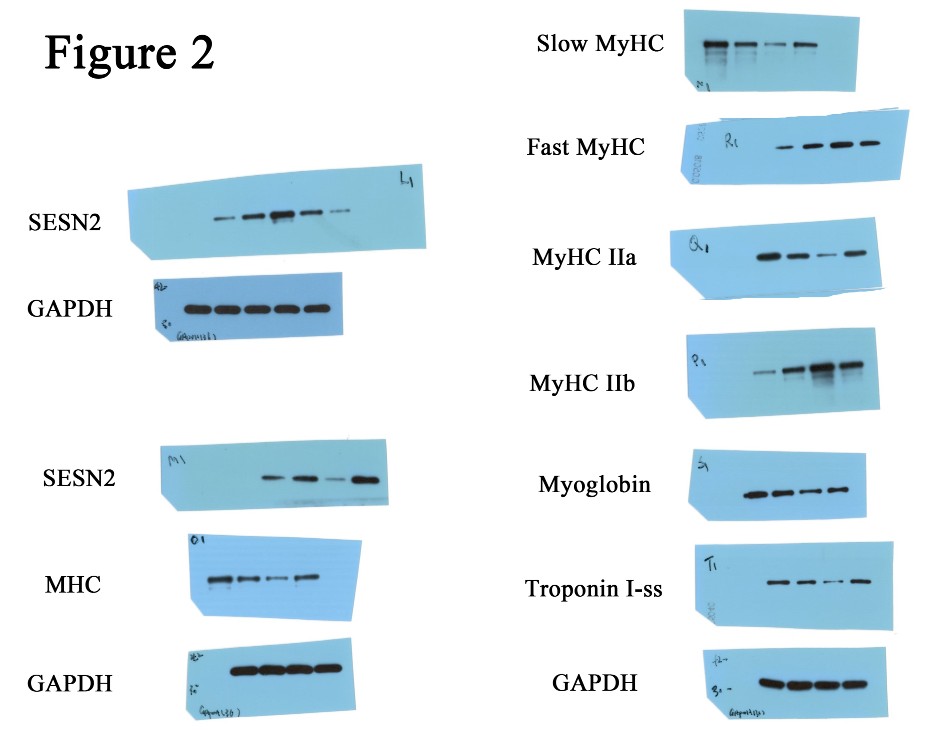


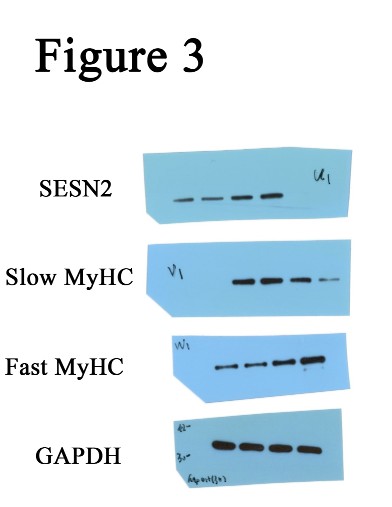


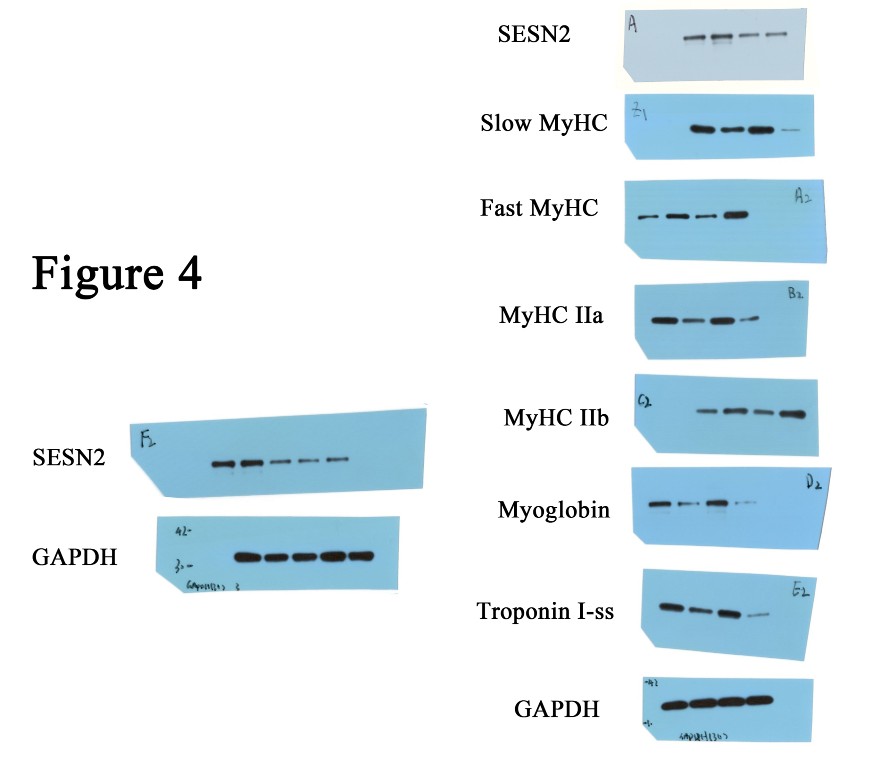


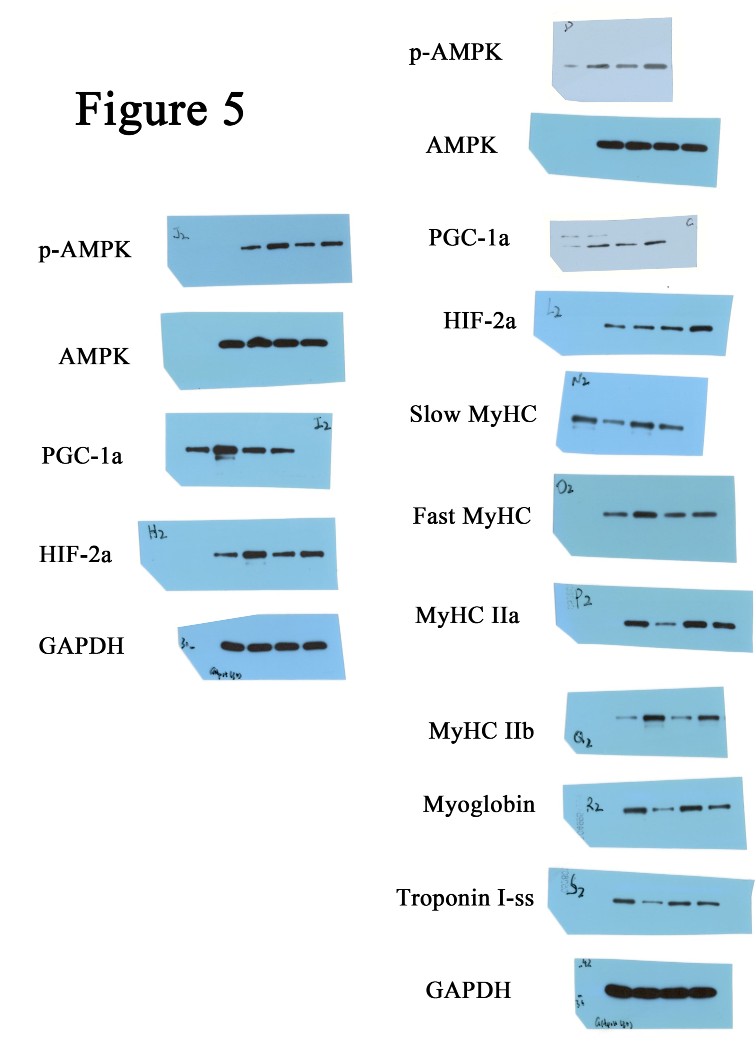


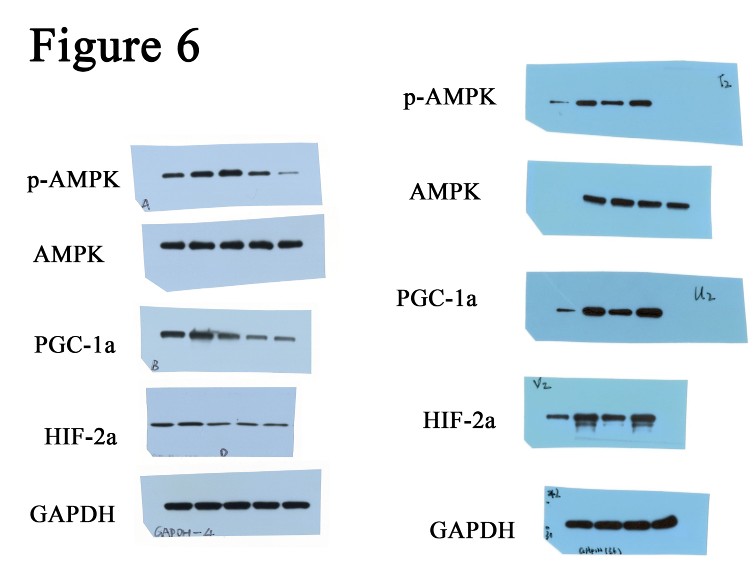

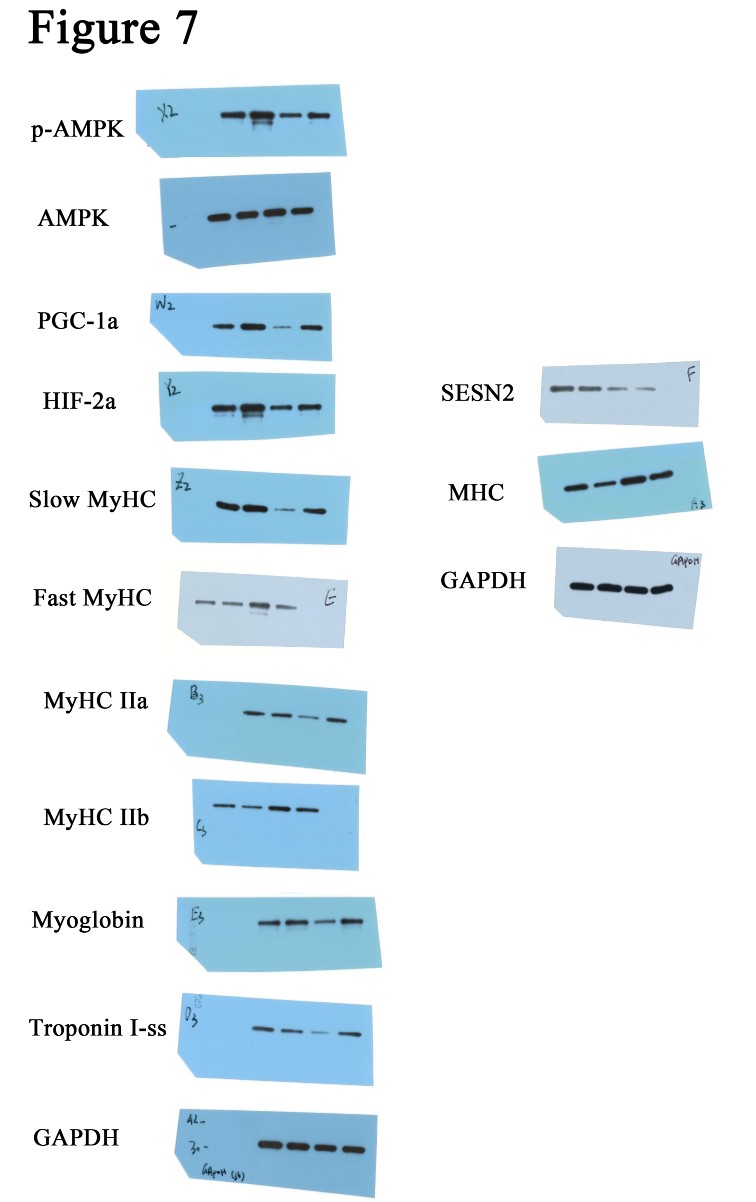

Supplement: Supplementary file 1 — Additional file 1: Table S1. The sequences of siRNAs. [file 11658_2022_367_MOESM1_ESM.docx]
